# Specialized Pro-Resolving Mediators Reduce Scarring After Cleft Lip Repair

**DOI:** 10.3389/fimmu.2022.871200

**Published:** 2022-04-27

**Authors:** Evangelos Papathanasiou, Andrew R. Scott, Carroll Ann Trotman, Corinna Beale, Lori Lyn Price, Gordon S. Huggins, Yang Zhang, Irene Georgakoudi, Thomas E. Van Dyke

**Affiliations:** ^1^Department of Periodontology, Tufts University School of Dental Medicine, Boston, MA, United States; ^2^Center for Clinical and Translational Research, Forsyth Institute, Cambridge, MA, United States; ^3^Department of Otolaryngology – Head & Neck Surgery, Tufts University School of Medicine, Boston, MA, United States; ^4^College of Dentistry, The Ohio State University, Columbus, OH, United States; ^5^Tufts Comparative Medicine Services, Tufts University, Boston, MA, United States; ^6^Tufts Clinical and Translational Science Institute, Tufts University, Boston, MA, United States; ^7^Institute of Clinical Research and Health Policy Studies, Tufts Medical Center, Boston, MA, United States; ^8^Molecular Cardiology Research Institute and Cardiology Division, Tufts Medical Center and Tufts University School of Medicine, Boston, MA, United States; ^9^Department of Biomedical Engineering, Tufts University School of Engineering, Medford, MA, United States; ^10^Department of Oral Medicine, Infection and Immunity, Faculty of Medicine, Harvard University, Boston, MA, United States

**Keywords:** scar, inflammation, cleft lip, lipoxins, collagen, rabbit, wound healing, surgery

## Abstract

**Objective:**

Residual scarring after cleft lip repair surgery remains a challenge for both surgeons and patients and novel therapeutics are critically needed. The objective of this preclinical experimental study was to evaluate the impact of the methyl-ester of pro-resolving lipid mediator lipoxin A_4_ (LXA_4_-ME) on scarring in a novel rabbit model of cleft lip repair.

**Methods:**

A defect of the lip was surgically created and repaired in eight six-week old New Zealand white rabbits to simulate human cleft lip scars. Rabbits were randomly assigned to topical application of PBS (control) or 1 ug of LXA_4_-ME (treatment). 42 days post surgery all animals were euthanized. Photographs of the cleft lip area defect and histologic specimens were evaluated. Multiple scar assessment scales were used to compare scarring.

**Results:**

Animals treated with LXA_4_-ME exhibited lower Visual Scar Assessment scores compared to animals treated with PBS. Treatment with LXA_4_-ME resulted in a significant reduction of inflammatory cell infiltrate and density of collagen fibers. Control animals showed reduced 2D directional variance (orientation) of collagen fibers compared to animals treated with LXA_4_-ME demonstrating thicker and more parallel collagen fibers, consistent with scar tissue.

**Conclusions:**

These data suggest that LXA_4_-ME limits scarring after cleft lip repair and improves wound healing outcomes in rabbits favoring the resolution of inflammation. Further studies are needed to explore the mechanisms that underlie the positive therapeutic impact of LXA_4_-ME on scarring to set the stage for future human clinical trials of LXA_4_-ME for scar prevention or treatment after cleft lip repair.

## Highlights

Specialized Pro-resolving Mediator (SPM) LXA_4_-ME limits residual scar tissue formation after cleft lip repair and improves wound healing outcomes in rabbits by favoring the resolution of inflammation.

## Introduction

Cleft lip with or without cleft palate is the most common congenital malformation of the head and the third most common birth defect (1). Surgical repair is the only treatment for cleft lip and usually happens during the early months of life. However, residual scar tissue formation is a frequent postoperative complication of cleft lip repair that impairs soft tissue form, function or movement, and also facial growth (2). Scar tissue generally occurs within 3-6 months following the initial surgery with a wide prevalence range from 8% to 47% (2, 3). Many patients (and caregivers) are dissatisfied with the surgical results, and multiple lip revision surgeries are required throughout childhood to optimize esthetics and function, usually between 5 and 8 years of age or later during adolescence (4). Cleft lip surgical revisions cause increased parental and patient stress, added anesthetic and surgical risks, and increased financial and societal costs (2).

Numerous studies have implicated excessive, persistent inflammation as detrimental to proper healing and an integral component of fibrosis and scar tissue formation (5–7). Histologic analysis of scar tissue has demonstrated an increased number of neutrophils, fibroblasts and myofibroblasts in scar tissue compared with normal skin healing lesions (7, 8). Scar tissue also contains dense parallel-oriented collagen fibers with perpendicularly- oriented capillaries, diminished hyaluronic acid content, and nearly absent to very sparse elastic fibers (9). Excessive production of pro-inflammatory cytokines disrupts normal wound healing and results in fibrosis. Thus, several cytokines (e.g. IL-1β, TNF-α, IL-6), and growth factors (e.g. TGF-β) have been identified as potential targets of antifibrotic therapy, with limited success. In the face of uncontrolled host immune defense mechanisms, tissue engineering, regeneration, and reconstruction of both diseased and injured oral and craniofacial tissues are significantly hampered (10).

It is generally believed that if inflammation does not resolve, wound healing and regeneration will not occur (11). To our knowledge, there is currently no safe and effective medical approach to predictably prevent or eliminate scarring after cleft lip repair (12). Current therapeutic agents block inflammation during healing. Intra-lesion steroid injections, especially triamcinolone, combined with topical administration of corticosteroid creams, historically are the preferred treatment for scars, but varying success has been reported with significant side effects including granuloma formation and skin atrophy (13, 14). Thus, there is a critical need for novel non-invasive treatments for the prevention or elimination of unresolved scars (13, 15).

Orchestrated resolution of inflammation is crucial for restoration of homeostasis and tissue regeneration (16). More recent discoveries indicate that effective resolution of inflammation is an active biologic process driven by endogenous agonists, referred to as Specialized Pro-resolving Mediators (SPMs) (16–18). SPMs include lipoxins, aspirin-triggered lipoxins (ATLs), resolvins (RvE, RvD), protectins and maresins. SPMs are generated by enzymatic oxygenation of n-3 and n-6 polyunsaturated fatty acids (PUFAs) after the initial stages of the inflammatory cascade and bind to specific receptors to actively resolve acute inflammation (19). SPMs act through a feed-forward receptor-mediated mechanism to prevent tissue fibrosis while promoting natural scar remodeling that was blocked by excessive inflammation (15). Based on findings that an imbalance between pro-inflammatory and pro-resolving mediators contributes to chronic inflammation and fibrosis, impaired inflammation resolution offers a mechanistic rationale for SPM therapy to enhance resolution of inflammation and promote natural, uninterrupted tissue remodeling (15, 20, 21).

SPMs can foster wound healing and tissue regeneration through their anti-inflammatory and pro-resolving actions by limiting neutrophil influx and activity, improving phagocytosis by macrophages and decreasing pro-inflammatory cytokine production, stimulating the clearance of inflammatory debris and promoting the return to tissue homeostasis (22, 23). SPMs can also modulate T-cell responses by decreasing their proinflammatory activities and promoting Treg cells, supporting SPM-based therapy as a promising avenue for the treatment of a wide range of T-cell-mediated immune and autoimmune diseases (24). SPMs can also mediate the crosstalk between glial cells and neurons suggesting new therapeutic strategies for different diseases of the CNS that need to be explored further (25). Topical application of an LXA_4_ analog in children was first successfully used in infantile eczema (26). Emerging evidence that SPMs accelerate wound healing in several tissues such as diabetic wounds (27), corneal wound healing (28) and skin fibrosis (17, 29) opens new opportunities for therapeutic approaches in the prevention and management of scarring in the orofacial region and can revolutionize wound healing outcomes.

This preclinical experimental study assessed the therapeutic impact of a well characterized SPM, the methyl-ester of lipoxin A_4_ (LXA_4_-ME) on scar formation after cleft lip repair using a recently established animal model of scarring (30).

## Materials and Methods

### Experimental Design

#### Animals

A total of 8 six-week old male New Zealand White rabbits weighing between two and three kilograms (kg) were purchased from Charles River Laboratories (MA, USA) and acclimatized 7 days before surgical procedures. All animals were kept in individual cages, received water *ad libitum*, and were fed standard rabbit chow at the Division of Laboratory Animal Medicine (DLAM), Tufts Medical Center (Boston, MA, U.S.A). Once weekly, the animals were weighed to ensure proper growth and nutrition. All experiments were approved by the Tufts University Institutional Animal Care and Use Committee (#B2017-58). All rabbit health, monitoring, husbandry, and experimental procedures were carried out in full accordance with standards set forth in the Guide for the Care and Use of Laboratory Animals, 8th edition (NIH Publication No. 85-23) and adequate measures were taken to minimize pain and discomfort for the animals. This study conformed to ARRIVE guidelines for preclinical animal studies.

#### Surgical Procedure

**Figure 1:**
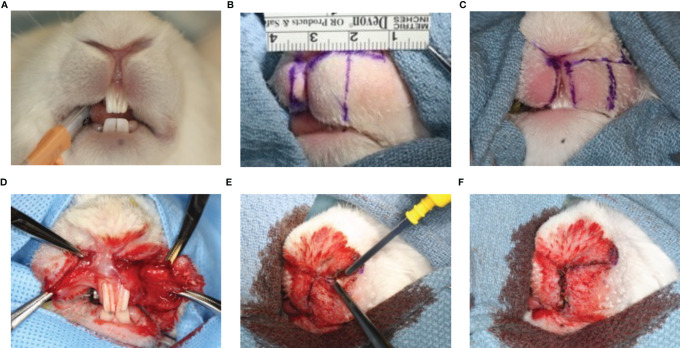
Animal model of scarring after cleft lip repair. Anesthesia was maintained with isoflurane 1-3% *via* endotracheal tube **(A)**. The upper lip on the left side was divided into two equal portions. The length of the upper lip of the left side was measured as the distance from the anterior border of the buccal pouch to the median line **(B)**. The medial portion of the upper lip on the left side was excised and a secondary releasing incision parallel to the upper lip on the left side was made **(C)**. A standardized rotation-advancement closure of the surgically induced cleft lip defect of the rabbit was performed with a passive adaptation of the flap **(D)**. The superficial skin edges were left open and lightly cauterized with electrocautery (also known as “buttering”) to assure hemostasis and encourage scarring **(E, F)**.

Prior to surgery, rabbits were anesthetized with an intramuscular mixture of ketamine (30 mg/kg) and Xylazine (5 mg/kg) and with isoflurane 1-5% *via* mask for induction. Rabbits were secured in the Trendelenburg position to avoid aspiration and anesthesia was maintained with isoflurane 1-3% *via* endotracheal tube (**Figure 1A**). We used a feasible and safe animal model for creating scarring after rotation-advancement cleft lip repair that we previously established (30). While anesthetized, the lips were shaved and a cream containing calcium hydroxide (Nair^®^, Church & Dwight) was applied. A defect of the lip was surgically created to simulate a unilateral cleft lip and then repaired, as originally described by Bardach et al. (31) with modifications (30). The upper lip on the left side was divided into two equal portions. The length of the upper lip of the left side was measured as the distance from the anterior border of the buccal pouch to the median line (**Figure 1B**). The medial portion of the upper lip on the left side was excised with surgical scissors and a blade (triangular in shape and extended both horizontally and vertically), creating a full thickness standardized defect of the lip of 100 mm^2^ and equal to one half of its normal width (which is approximately 25 mm). A secondary releasing incision parallel to the upper lip on the left side as a 10 mm, lateral back-cut was planned with a small Burow’s triangle to decrease the tension of the flap, allow its release and facilitate closure. The edges of the medial portion of the upper right half lip were marked and slightly excised in order to allow for better adaptation with the excised upper half lip (fresh-to-fresh wound edges) (**Figure 1C**). A standardized rotation-advancement closure of the surgically induced cleft lip defect of the rabbit was performed with a passive adaptation of the flap (**Figure 1D**). In order to induce sufficient scarring, we closed the lip defect using 6 interrupted/subcuticular 4-0 absorbable sutures (Chromic gut) for underlying mucosal layer and 6 interrupted/subcuticular/dermal 3-0 non-absorbable suture (silk) for the muscle and deep dermal layer. The superficial skin edges were left open and lightly cauterized with electrocautery (also known as “buttering”) to assure hemostasis and encourage scarring (**Figures 1E, F**).

#### Treatment

Eight rabbits were randomly assigned to two different experimental groups: Group I (Treatment): Four animals with surgically induced cleft lip defects were repaired and 100 μl of a 10 μg/ml of LXA_4_-ME (1.0 µg/ml) diluted in phosphate-buffered saline (PBS) was topically applied with pipettes. Group II (Control): Four animals received topical application of 100 μl of PBS (vehicle) with pipettes after cleft lip surgical repair.

We followed a simple randomization approach for assigning the animals to control or treatment group. The application of either LXA_4_-ME (Cayman Chemical, Ann Arbor, MI) or PBS was immediately after cleft lip repair surgery and on a daily basis for the first week and three times/week from second week until time of euthanasia (Monday-Wednesday-Friday). The dose of 1.0 µg/ml of LXA_4_-ME and the application schedule were selected based on previous successful topical application of LXA_4_ in other animal trials (32, 33) and on a pilot testing of two different doses of LXA_4_-ME (0.1 µg/ml or 1.0 µg/ml).

#### Postoperative Care and Euthanasia

Antibiotics (Baytril 5mg/kg SQ at time of surgery and once a day for 3 days) were administered to all animals to prevent infection. All animals were fed with soft critical care diet for the first 3-4 days after surgery and then with standard rabbit chow. Animals were monitored three times/day for the first three days and then once daily for the remaining experimental period. Before euthanasia, macroscopic pictures of the wound area were taken with a digital camera and were used for Visual Scar Assessment as described below. Animals were euthanized with intravenous overdose injection (100 mg/kg) of sodium pentobarbital (i.e., Euthasol) in the peripheral ear vein 42 days post-operatively. Death was confirmed by lack of heartbeat and ECG. This method is listed as an approved method in the AVMA Guidelines on Euthanasia (2013 edition).

After euthanasia of animals, the wound area and surrounding tissue samples around the repaired cleft lip defect were excised and fixed in 4% paraformaldehyde. Sections were stained with hematoxylin-eosin (H&E) and with Masson’s Trichrome Stain, and were used for scar assessment analysis and comparisons between the two experimental groups.

#### Studied Outcomes

a) Visual Scar Assessment: Before euthanasia, macroscopic high resolution photographs of the wound area of the surgically repaired cleft lip defect were reviewed and graded independently by two different examiners blinded to the status of each rabbit. Each examiner reviewed and independently graded the clinical scars using the Modified Manchester Scar Scale (34, 35). The Modified Manchester Scar Scale is displayed in **Supplementary Figure 1** (**Appendix**). The score range was from 5 (best possible scar) to 26 (worst possible scar) for each rabbit. Each examiner conducted two rounds of grading of the photographs, separated by at least one week, and entered the scores for each rabbit in Excel Spreadsheets (Microsoft ®).
b) Histology: Tissue samples previously fixed in 4% paraformaldehyde at 4°C for 48 hours were dehydrated through a series of ethanol washes, cleared in xylene, embedded in paraffin, and 5μm thick sections cut (36). The sections were deparaffinized in xylene, re-hydrated through a series of ethanol washes and stained with H&E or with Masson’s Trichrome stain in order to assess inflammatory cell infiltration and collagen density and organization, respectively. JPEG images of mounted sections were captured at 20x, 40x, 100x and 400x magnification with a digital microscope (Zeiss Axio Observer A1) equipped with a digital camera (AxioCam HRc r1.6) (37).

Examination of histologic specimen: At 20x magnification the suture line of the surgically repaired cleft lip defect was identified. The apico-coronal (lower-upper) length of the specimen (approximate length of 4.8 mm) was measured (EP) using Aperio Imagescope software (Leica Biosystems Imaging, Inc, CA, USA) and divided in three equal regions (apical, middle, coronal) with boxes of equal standardized dimensions (3 x 1.5 mm). The coronal surface of the coronal region of the specimen was identified at the level of the outer epithelial surface and extended 1.5 mm in the apical direction and 1.5 mm away from the suture line. The coronal surface of the middle region of the specimen was marked at a distance of 3.0 mm from the outer epithelial surface. The coronal surface of the apical region of the specimen was marked at a distance of 4.5 mm from the outer epithelial surface.

JPEG images of each region (apical, middle, coronal) were captured for each rabbit at 100x, saved and then distributed to both examiners (EP,TVD) for further analysis and grading.

Histologic Scar Assessment (Subjective): 1 image of each region of the specimen (coronal, middle, apical) at 100x magnification from each rabbit (3 regions/rabbit) were used for review and grading (total n=24 images). All images were reviewed and graded independently by two different examiners (EP,TVD) using the Histologic Scar Assessment scale described in **Supplementary Figure 2** (**Appendix**). Each examiner conducted two rounds of grading of the images separated by at least one week.

Inflammation was scored as 0 (none), 1 (mild), 2 (moderate), or 3 (severe) (38, 39). Scar formation in the connective tissue was graded based on collagen fiber bundle density (inter-fiber bundle space) with a score of 0 (normal), 1 (mild), 2 (moderate), or 3 (severe) (score of 0 indicates no difference from unwounded/normal tissue) (40, 41). The score given for each histologic image of the wound area can range from 0 (best possible scar/normal) to 6 (worst possible scar). Each examiner calculated the total score for each region of the histologic specimen and the mean score of the Histology Scar Assessment of three regions (apical, middle, coronal) of each rabbit specimen in Excel Spreadsheets (Microsoft ®).

#### Automated Quantitative Scar Assessment:

i) The density of inflammatory cell infiltrate from H&E-stained sections was determined using the Nuclear image analysis algorithm (Nuclear V9) of Aperio Imagescope software (Leica Biosystems Imaging, Inc, CA, USA). Briefly, inflammatory cells were clearly distinguishable based on morphologic characteristics and enumerated using the Nuclear image analysis algorithm at 400x magnification. The parameters of the Nuclear image analysis algorithm were first adjusted using “algorithm tuning” in a mark-up window to ensure that all nuclei are properly segmented and identified. As soon as the parameters of the Nuclear image analysis algorithm were adjusted, the same parameters were applied for the analysis of all images. Each region of analysis had the same total surface. The average cell density (cells/sqmm) from 3 different regions of analysis from each rabbit specimen was automatically calculated and the results were exported in an Excel spreadsheet (Microsoft ®). Then, the average score of the cell density of three regions (apical, middle, coronal) of analysis from each specimen was calculated (please see **Supplementary Figure 3** in **Appendix**).
ii) An automated quantification of collagen fiber density and 2D directional variance (orientation) of collagen fibers from Masson’s Trichrome stained sections of rabbit tissues 42 days after cleft lip repair was also conducted. RGB images of Masson’s Trichrome Stained tissue sections from each region of the specimen (apical, middle, coronal) were provided to the laboratory of Dr. Irene Georgakoudi (Tufts School of Engineering, Medford, MA). Each RGB image of Masson’s Trichrome Stained tissue sections was in JPEG format and analyzed using custom-written code in Matlab (Mathworks, Natick, MA, USA) adapted from an approach to assess collagen fiber density and orientation following previously described protocols (42) (see **Supplementary Figure 4** in **Appendix**). Since collagen fibers are stained blue, they are identified most clearly by a decrease in the red channel transmission, when compared to other features like elastin, fur, blood vessels, and epithelial cells. For this reason, Otsu’s global thresholding was applied to the red channel to separate the pixels to four different intensity levels and retain the pixels with values in the two lower intensity levels. From these pixels, the ones with ratio values less than 2.5 in the B/G map were kept, and a second Otsu’s four level thresholding was applied on the remaining pixels of R/B images, in order to separate the pixels in the lowest intensity threshold. To minimize computational time, all images were automatically rotated and cropped to include only the collagen region of the histologic sections. The remaining area represented consistently the collagen fiber regions of the slides and we used the intensities in the red channel to calculate the fiber orientation at each pixel using a weighted alignment vector summation technique within a 5x5 pixel window (2.3x2.3μm^2^). The 2D directional variance was computed as a quantitative metric of fiber alignment at each pixel within a 3-pixel radius disk kernel filter. The 2D directional variance value varies between 0 and 1, corresponding to perfectly aligned and disorganized collagen fibers, respectively (43). The collagen fiber density within each field was calculated based on the relative number of collagen-containing pixels compared to the total field pixel number (i.e. excluding blank areas). All images were analyzed by one investigator (YZ), who was blinded to the study treatment assignment of the animals.

Sample size calculation: To achieve 80.0% power to reject the null hypothesis of equal means of collagen fiber density (%) when the population mean difference is 0.28 with a standard deviation (SD) for both groups of 0.10 and with a significance level (alpha) of 0.050 using a two-sided two-sample equal-variance t-test, 4 animals per group were required. We consider a population mean difference of 28.0% to be a clinically meaningful difference because cleft lip surgeons typically view a reduction of scar tissue (size, extent) of at least 25% to be a clinically meaningful improvement in patients undergoing cleft lip repair surgery (12, 44, 45).

#### Statistical Analysis

A 2-way random-effects ANOVA model was used to calculate estimates of intraclass correlation coefficient (ICCs) and their 95% confidence intervals (CI) for Modified Manchester Scar, while kappa statistics were calculated for Histologic Scar assessment (46). We also constructed line of identity plots and Bland-Altman plots (47) in order to allow visual assessment of the magnitude of agreement/disagreement between the two examiners and the two rounds of measurements for Modified Manchester Scar and to identify potential systematic bias. We calculated Spearman’s rank correlation coefficients between Modified Manchester Scar and mean of three regions for Histologic Scar Assessment Scale and explored their associations by constructing scatter plots.

We used the Mann-Whitney U test to compare the Modified Manchester Scar and the Histologic Scar Assessment scores, the density of inflammatory cells (cells/sqmm) and collagen fiber density (%) between Control and Treatment groups. For this comparison we used the Modified Manchester Scar score and the mean score of the Histologic Scar Assessment Scale of three regions (apical, middle, coronal) of each histologic specimen from the first round of grading from one examiner only (reference examiner) (TVD) who was blinded to the study treatment assignment of the animals. We used the Rstudio software for our data analysis and the SPSS software (version 27.0) for the construction of the boxplots displayed in the figures. Results were considered significant at p < 0.05.

## Results

### LXA_4_-ME Reduces Visual Scarring

In our preclinical experimental study in a novel rabbit model of cleft lip repair (**Figure 1**), the topical application of LXA_4_-ME after cleft lip repair surgery in rabbits resulted in an improvement in wound healing and reduction in visual scarring assessed with the Modified Manchester Scar Scale compared to topical application of PBS (n=4/group). Macroscopic images (JPEG) of the wound area captured before euthanasia were evaluated by one examiner (TVD) who was blinded to the study treatment assignment of the animals. The intra-rater reliability for our reference blinded examiner (TVD) for Modified Manchester Scar was excellent with an ICC of 0.97 (95% CI=0.90 to 0.99) (n=8, 1 image/rabbit) (see also Apendix for more details). Based on Bland-Altman plot and line of identity plot analyses (**Supplementary Figures 5**, **6** in **Appendix**), there is lack of evidence of systematic bias in the reported Modified Manchester Scar scores.

**Figure 2:**
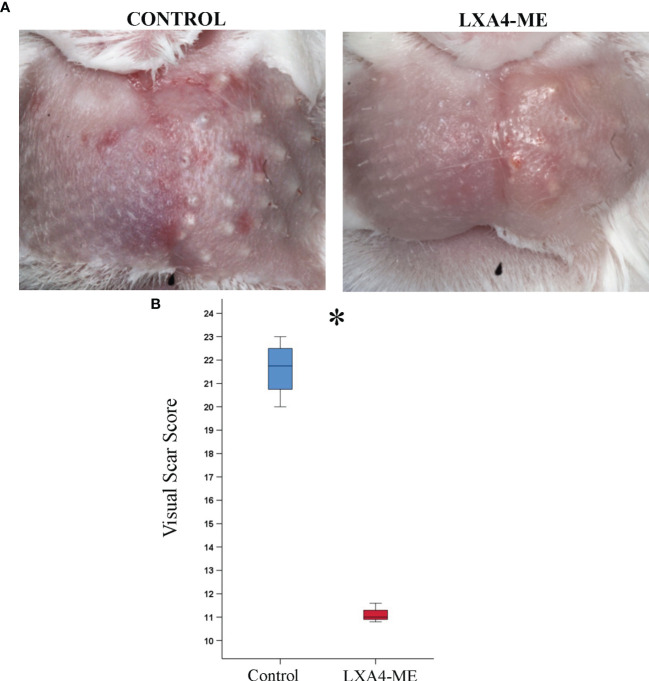
Visual Scar Assessment. **(A)** Clinical photographs 42 days after cleft lip repair surgery in rabbits treated with phosphate-buffered saline (PBS) (control) or with methyl-ester of lipoxin A_4_ (LXA_4_-ME) (treatment). **(B)** Animals treated with LXA_4_-ME demonstrated reduced visual scarring scores assessed with the Modified Manchester Scar Scale (median=11.00; 25^th^; 75^th^ percentile=10.95, 11.15) compared to animals that received only PBS (control) (median=21.75; 25^th^; 75^th^ percentile=21.12, 22.25) (statistically significant at *P < 0.05, Mann-Whitney U test; n=4 animals/group).

The clinical visual assessment of animals treated with LXA_4_-ME demonstrated an improved wound healing outcome 42 days after cleft lip repair surgery with reduced redness and edema and smaller spread of scarring compared to control animals (**Figure 2A**). In addition, animals treated with LXA_4_-ME showed decreased distortion of operated side of the upper lip compared to adjacent non-operated tissue suggesting a more desirable overall impression compared to control animals. Statistical analysis of visual scar assessment (Mann-Whitney U test; p=0.029) confirmed that the animals treated with LXA_4_-ME received significantly lower scores of the Modified Manchester Scar Scale (median=11.00; 25^th^; 75^th^ percentile=10.95, 11.15) indicating reduced scarring compared to control animals (median=21.75; 25^th^; 75^th^ percentile=21.12, 22.25) (**Figure 2B**).

### LXA4-ME Reduces Inflammatory Cells

Qualitative and quantitative analysis of histologic specimens of a total of 8 rabbits 42 days after cleft lip repair surgery was conducted. The intra-rater reliability for our reference blinded examiner (TVD) for Histologic Scar assessment of 24 images (2 rounds of 3 regions for 8 rabbits x 1 image/region) stained with H&E was good with a range of weighted kappa statistics from 0.67 to 1.00 (see also Apendix for more details).

**Figure 3:**
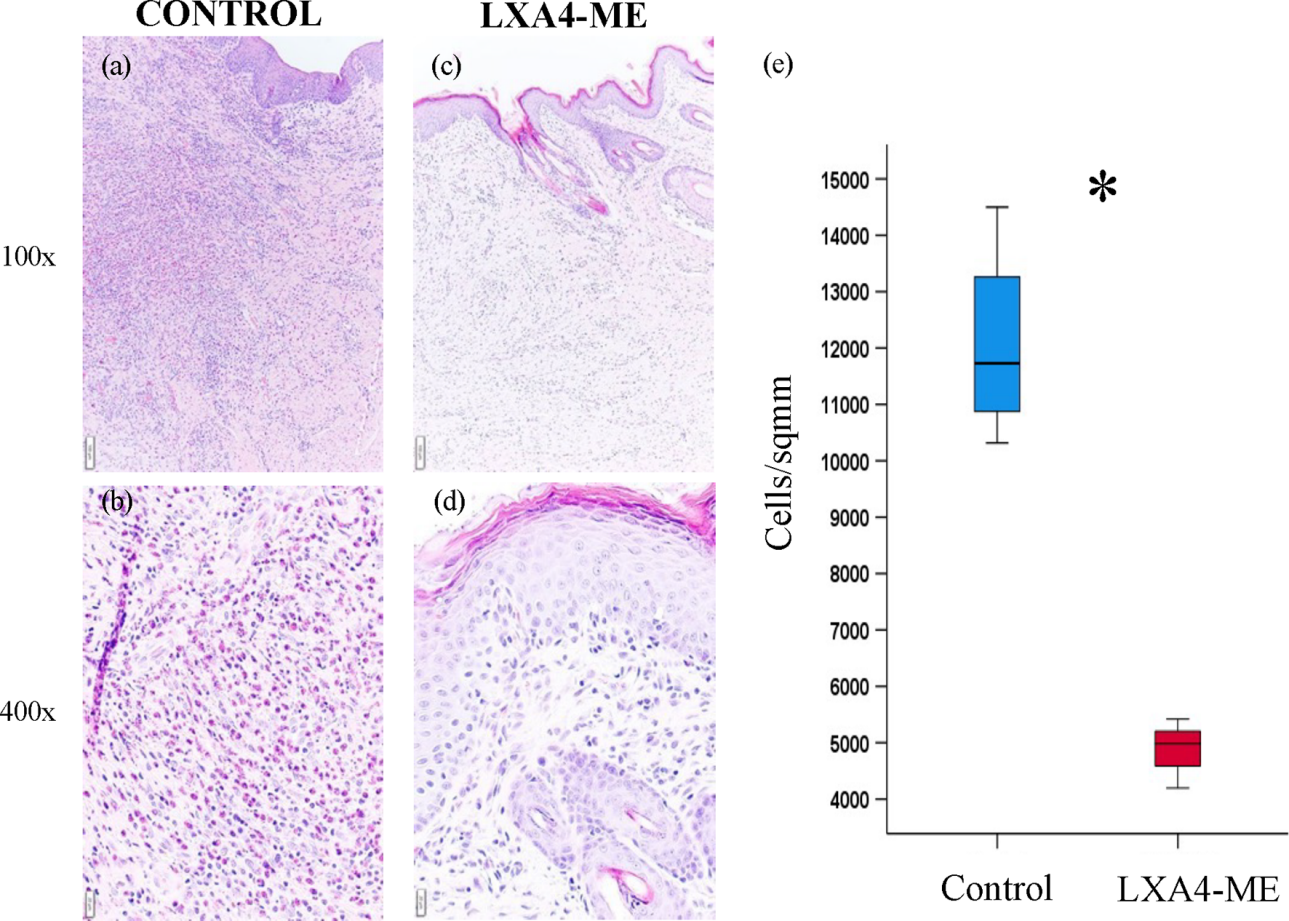
Microscopic examination of images of representative tissue specimens stained with hematoxylin and eosin (H&E) captured at 100x **(A, C)** and 400x **(B, D)** magnification. The scale bars represent 100 um **(A, C)** and 20 um **(B, D)**. Automated quantification of inflammatory cell infiltrate with Aperio Imagescope software confirmed significant reduction of inflammatory cell density in animals treated with methyl-ester of lipoxin A_4_ (LXA_4_-ME) (median=4986; 25^th^; 75^th^ percentile=4784, 5099) compared to control ones treated with PBS (median=11730, 25^th^; 75^th^ percentile=11158, 12643) **(E)** (statistically significant at *P < 0.05, Mann-Whitney U test; n=4 animals/group). The averages of three regions per animal were used for analysis.

Analysis of histological sections (n=12/group) stained with H&E showed reduced inflammatory cell infiltrates in animals treated with topical application of LXA_4_-ME after cleft lip repair surgery (**Figures 3C, D**) compared to control treated with PBS (**Figures 3A, B**). Treatment with LXA_4_-ME after cleft lip repair resulted in a significant reduction in inflammatory cell infiltrates (cells/sqmm) (median=4986; 25^th^; 75^th^ percentile=4784, 5099) that were quantified by one blinded examiner (EP) with Aperio Imagescope software (Leica Biosystems Imaging, Inc, CA, USA) compared to PBS application (median=11730, 25^th^; 75^th^ percentile=11158, 12643) (p=0.029; Mann-Whitney U test; n=4 animals/group) (**Figure 3E**).

### LXA4-ME Reduces Collagen Fiber Density

**Figure 4:**
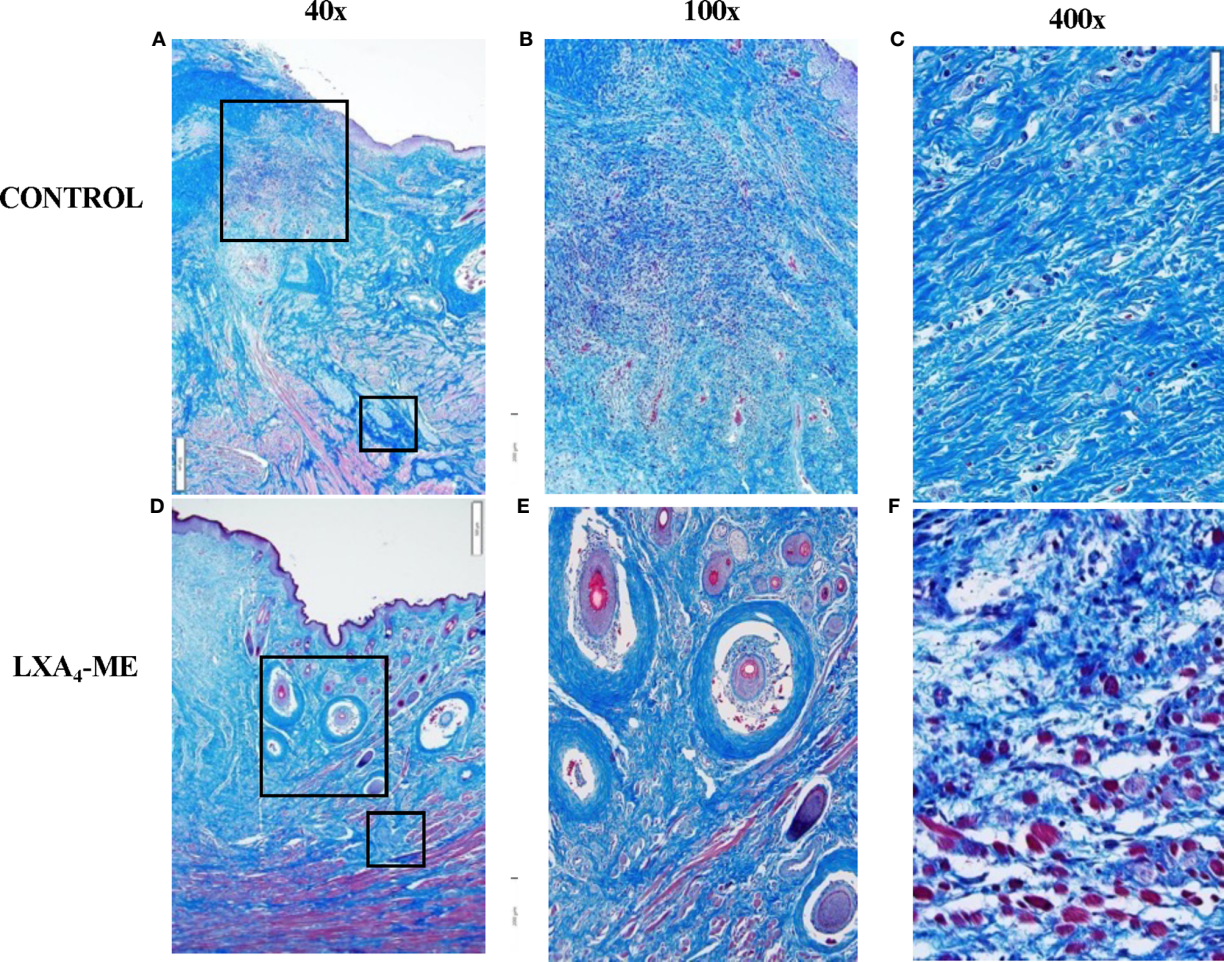
Microscopic examination of images of representative tissue specimens stained with Masson’s Trichrome captured at 40x magnification **(A, D)**. Large and small rectangular areas were captured at 100x **(B, E)** and 400x **(C, F)**, respectively. Animals treated with LXA_4_-ME after cleft lip repair showed decreased density of collagen fibers compared to control treated with PBS. A higher number of hair follicles was observed in histological sections of animals treated with LXA_4_-ME **(D, E)** compared to control **(A, B)**, indicating wound healing closer to normal skin than scar tissue. Muscle fibers and cells were more frequently identified within collagen fibers in animals treated with LXA_4_-ME **(D, F)**, while scar tissue and dense collagen interfered with muscle regeneration in control animals **(A,C)**. The scale bars represent 500 um **(A, D)**, 200 um **(B, E)** and 50 um **(C, F)**.

Analysis of histological sections stained with Masson’s Trichrome showed decreased density of collagen fibers in animals treated with topical application of LXA_4_-ME after cleft lip repair compared to control treated with PBS (**Figure 4**). The intra-rater reliability for our reference blinded examiner (TVD) for Histologic Scar assessment of 24 images (2 rounds of 3 regions for 8 rabbits x 1 image/region) stained with Masson’s Trichrome was good with a range of weighted kappa statistics from 0.75 to 0.84 (see also Apendix for more details).

A higher number of hair follicles was observed in histological sections of animals treated with LXA_4_-ME (**Figures 4D, E**) compared to control (**Figures 4A, B**), indicating wound healing closer to normal skin than scar tissue. A very interesting observation during analysis of histological sections stained with Masson’s Trichrome was that in animals treated with LXA_4_-ME muscle fibers and cells were more frequently identified within collagen fibers, indicating muscle regeneration after cleft lip repair surgery (**Figures 4D, F**). This finding was not present in control animals treated with PBS (**Figures 4A, C**), likely due to interference of scar tissue and dense collagen with muscle regeneration after cleft lip repair. Statistical analysis of histologic scar scores provided by one blinded examiner (TVD) (Mann-Whitney U test; p=0.027) confirmed that the animals treated with LXA_4_-ME received significantly lower scores with the Histology Scar Assessment Scale (median=2.17; 25^th^; 75^th^ percentile=2.00, 2.33), indicating reduced scar tissue formation compared to control animals (median=4.83; 25^th^; 75^th^ percentile=4.33, 5.00).

### LXA_4_-ME Improves Collagen Remodeling

**Figure 5:**
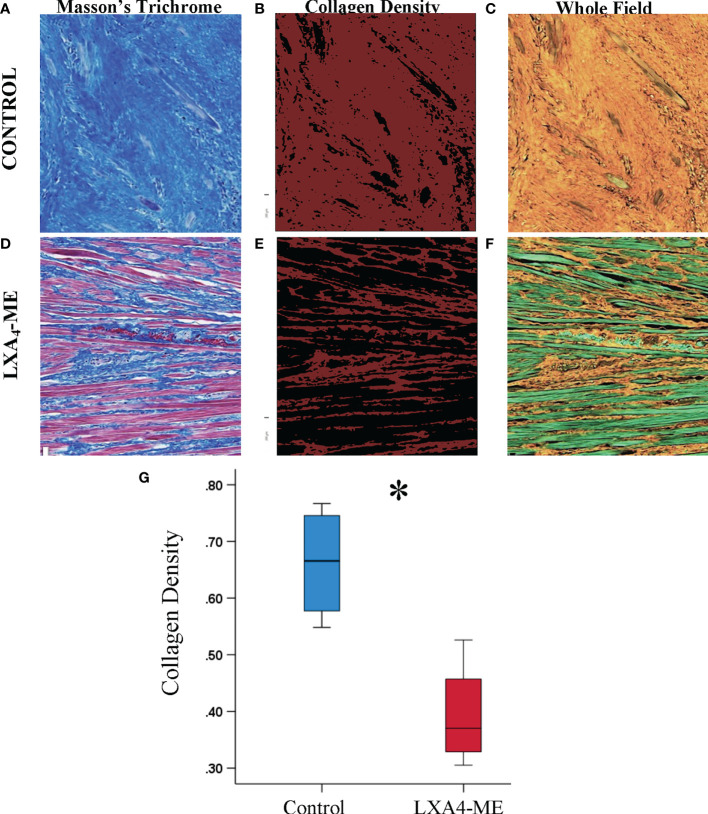
Automated quantification of collagen fiber density of JPEG images of representative tissue specimens stained with Masson’s Trichrome captured at 200x magnification **(A, D)**. Collagen fibers are coded red in images illustrating collagen density **(B, E)** or orange in whole field images **(C, F)**. Automated quantification of collagen fiber density using custom-written code in Matlab showed reduced collagen fiber density in animals treated with LXA4-ME (median=0.37, 25^th^; 75^th^ percentile=0.34, 0.42) **(E, F)** compared to control animals treated with PBS (median=0.67, 25^th^; 75^th^ percentile=0.59, 0.74) **(B, C)**. The average of three regions per animal were used for statistical analysis **(G)** (*P < 0.05, Mann-Whitney U test; n=4 animals/group). The color bars for panel b and e are ranging from 0-1. The scale bars represent 100 um.

**Figure 6:**
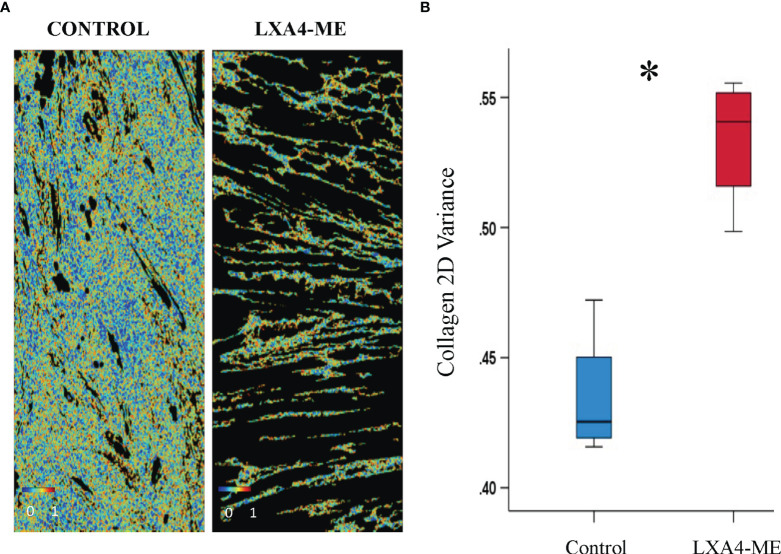
Automated quantification of 2D directional variance (orientation) of collagen fibers of JPEG images of representative tissue specimens stained with Masson’s Trichrome captured at 200x magnification. **(A)** Topical treatment with PBS in control animals resulted in reduced 2D directional variance (median=0.42, 25^th^; 75^th^ percentile=0.42, 0.44) compared to treatment with LXA_4_-ME (median=0.54, 25^th^; 75^th^ percentile=0.52, 0.55) **(B)** (*P < 0.05, Mann-Whitney U test; n=4 animals/group). The average of three regions per animal were used for analysis. The color bars are ranging from 0-1.

In addition to subjective scoring of collagen density we also used an automated pixel-wise analysis of fiber orientation within histology images to quantify the density and 2D variance (orientation) of collagen fibers. Masson’s Trichrome stained sections (**Figures 5A, D**) (JPEG images) of rabbit tissues 42 days after cleft lip repair were automatically analyzed by one blinded examiner (YZ) using algorithms that enable quantification of collagen fiber density (**Figures 5B, C, E, F**) and **Figure 2D** directional variance (orientation) (**Figure 6**). Animals treated with LXA_4_-ME after cleft lip repair showed reduced collagen fiber density (%) (**Figures 5E, F**) (median=0.37, 25^th^; 75^th^ percentile=0.34, 0.42) compared to controls (**Figures 5B, C**) (median=0.67, 25^th^; 75^th^ percentile=0.59, 0.74) (p=0.029; Mann-Whitney U test; n=4 animals/group) (**Figure 5G**).

In addition, control animals treated with PBS showed reduced 2D directional variance (orientation) (%) of collagen fibers (median=0.42, 25^th^; 75^th^ percentile=0.42, 0.44) compared to animals treated with LXA_4_-ME (median=0.54, 25^th^; 75^th^ percentile=0.52, 0.55). This finding is consistent with the presence of collagen fibers that are more aligned with respect to each other in the control (PBS) group, which more closely resembles collagen orientation in scar tissue than normal skin collagen fibers (**Figure 6**) (p=0.029; Mann-Whitney U test; n=4 animals/group).

## Discussion

**Figure 7:**
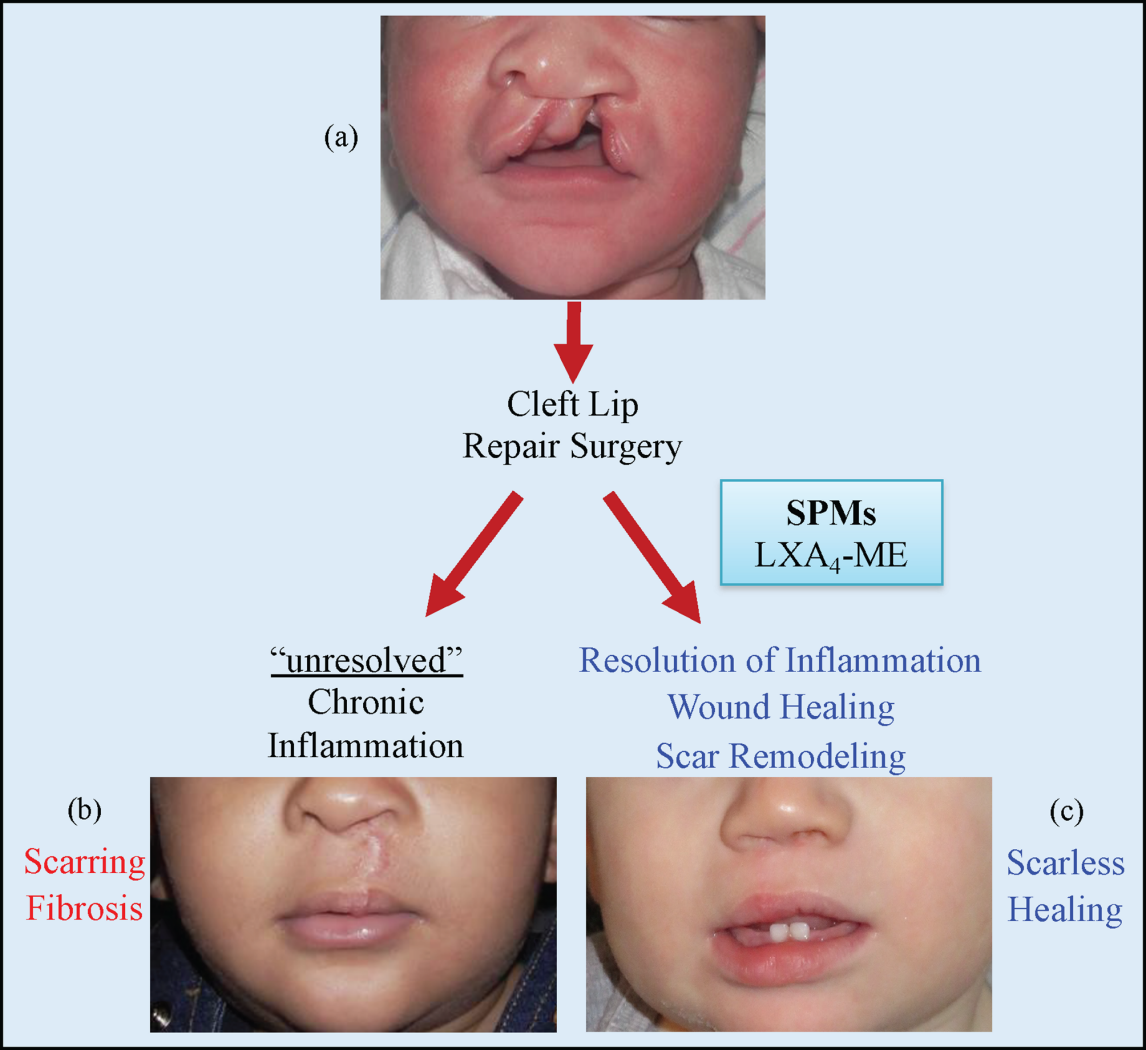
Cleft lip **(A)** is the most common congenital malformation of the head and the third most common birth defect. Surgical repair is the only treatment for cleft lip and usually happens during the early months of life. Failure of resolution of acute inflammation after cleft lip repair surgery will lead to chronic inflammation with scarring and fibrosis **(B)**. Modulation of inflammatory resolution pathways in acute and chronic wounds *via* the topical application of LXA_4_-ME and other Specialized Pro-resolving Mediators (SPMs) can optimize wound healing **(C)** and is a promising therapeutic avenue to reduce scarring after cleft lip repair surgeries and other surgical wounds.

The current report demonstrates that topical application of LXA_4_-ME reduces scar tissue formation after cleft lip repair surgery in a rabbit model. We show here that animals treated with LXA_4_-ME after cleft lip repair received lower scarring scores with the Visual Scar Assessment compared to animals treated only with PBS. In addition, treatment with LXA_4_-ME after cleft lip repair resulted in a significant reduction of inflammatory cell infiltrate and density of collagen fibers compared to PBS application. Moreover, control animals showed reduced 2D directional variance (orientation) of collagen fibers compared to animals treated with LXA_4_-ME indicating that collagen fibers were thicker and more aligned, resembling more scar tissue than normal skin collagen fibers. These findings support our central hypothesis that SPMs (LXA_4_-ME) limit residual scar tissue formation after cleft lip repair by favoring the resolution of inflammation and improving wound healing outcomes (**Figure 7**). All scar assessment scales that were used in this preclinical experimental animal study showed good reliability and lack of evidence of systematic bias in grading scarring.

Surgical repair of cleft lip is the only method of treatment that has the potential to secure the continuity of tissues and restore function and esthetics. Scarring after cleft lip repair is a frequent postoperative complication that requires many patients and caregivers to elect multiple lip revision surgeries. Cleft lip scar management should be considered a constant element in the treatment plan of cleft lip patients (12). Development of novel non-invasive topical treatments, for the prevention or elimination of unresolved scars is critical as current therapies for the management of scarring after cleft lip repair are minimally effective. Dysregulated inflammatory/lipid mediator signaling, and impaired resolution of acute inflammation contribute to scarring (5–7). The classic triad of regenerative medicine (scaffold, cells, and soluble mediators) is insufficient because of our current inability to control inflammation during regeneration (7, 10, 48). Emerging evidence that different SPMs accelerate wound healing by preventing chronic inflammation and allowing uninterrupted tissue remodeling suggests new therapeutic opportunities in the prevention and management of postsurgical cleft lip scarring (15). Thus, there is a rational argument for the development of SPMs that enhance tissue regeneration by promoting resolution of inflammation in our clinical armamentarium (49). Novel therapies for treating cutaneous pathological scarring may be extrapolated from clinical trials targeting fibrosis with SPMs in other organs including lung, kidney, heart, and cornea (50, 51). Importantly, to our knowledge, no previous studies have evaluated orofacial skin scarring outcomes in relation to SPMs or related compounds.

We use for the first time a novel animal model of scarring after cleft lip repair to evaluate the therapeutic potential of new drugs, specifically of an SPM (LXA_4_-ME). The rabbit model is the model of choice for several reasons. First, the rabbit is one of the two FDA accepted animal models for scarring and its treatment. In addition, the size of the rabbit and the anatomy of the lip approximate human infants and allow easier simulation of a surgical repair of the cleft lip defect with predictable formation of residual scarring as we have recently shown (52). While several animal models of cutaneous scarring already exist, they mainly use excisional incisions on either dorsal dermis of rodents or on ears of rabbits (53, 54). However, these previous animal models do not simulate the respective human healing conditions of cleft lip repair that include the interference from mechanical forces and wound tension and the interaction between oral mucosa, muscle, and dermal tissues during tissue remodeling. Resection of a segment of the lateral lip element in our model allowed for establishment of wound tension, which better imitates the forces at play during human healing after cleft lip repair, and exposure to oral microflora. Moreover, the rabbit has been previously selected to investigate the therapeutic impact of SPMs (32, 33, 55, 56) as rabbits show a very similar pattern of regulation of eicosanoid production (57) and lipid mediator profile (including resolvins and lipoxins). Rabbits have also been previously used as experimental models for the pharmacokinetic analysis of NSAIDs (58, 59).

Overall, the gold standard in scar assessment is microscopic evaluation of histologic specimens. Histologic evaluation is feasible in animal research where tissue biopsies are collected, but almost impossible in humans where visual scar assessment scales are used. As the ultimate goal of animal research is to translate findings and apply them in humans, we used Visual Scar Assessment Scales that are mainly used in clinical practice. The reliability of scar assessment scales in animal studies has been rarely calculated and presented in published literature and the inclusion of reliability in this preclinical experimental study is a step forward. It is important to determine reliability for our measurements for all scar assessment scales used in order to optimize consistency and repeatability in measurements and minimize random measurement errors. It is desirable to use rigorous assessments with good measures of reliability and reproducibility. The intra-examiner and inter-examiner reliability of Visual and Histologic scar assessment scales in our study indicates that scar assessment scales used in this preclinical experimental study showed good reliability and lack of evidence of systematic bias. By directly comparing the Visual Scar Assessment Scale against the Histologic Scar Assessment Scale, we demonstrate strong correlation (construct validity), indicating strong potential for the Visual Scar Assessment Scale to be used as the primary scar assessment outcome in future animal and human therapeutic investigations.

Modulating the function and proliferation of fibroblasts and immune cells is crucial to promote wound repair and scar reduction (60, 61). We demonstrate here a novel application of SPMs for the reduction of orofacial scarring after cleft lip repair. Our choice of LXA_4_-ME as the SPM molecule was based on previous studies that show that the methyl-ester of LXA_4_ is more stable during storage and serves as a pro-drug formulation of the transcellular metabolite LXA_4;_ the ME dissociates in an inflammatory environment leaving the active free acid. Human clinical LXA_4_ trials are underway (17, 26, 27, 50). The dose of 1 µg of LXA_4_-ME was selected based on previous successful topical application of LXA_4_ in other animal trials (32, 33, 62) and on a pilot testing of two different doses of LXA_4_-ME (0.1 µg/ml or 1.0 µg/ml). Automated quantification of cell density in our animal study showed that topical application of LXA_4_-ME reduced inflammatory cell infiltrate after cleft lip repair confirming histologic scores of subjective qualitative grading of inflammation (TVD) and findings from other similar studies of SPMs on wound healing (27, 63).

The gold standard method to determine collagen fiber density and direction in scar tissue relies on qualitative descriptions or subjective scoring systems from Masson’s trichrome stained histological sections. Automated pixel-wise analysis of fiber orientation within histology images enabled us to quantify the density and 2D variance (orientation) of collagen fibers. This is the first time that this novel methodology has been used to evaluate orofacial scarring and wound healing. This analysis technique offers a straightforward approach to track tissue remodeling and provide objective surrogate endpoints for evaluating the collagen organization of scar tissue. LXA_4_ and RvD2 have been shown to modulate fibroblast proliferation and migration directly to limit fibrosis at early time points, allowing wound healing and collagen deposition in the ECM to proceed normally (17). Automated quantification of collagen fiber density showed reduced collagen fiber density in animals treated with LXA_4_-ME compared to control treated with PBS, confirming histologic scores of subjective qualitative grading of collagen density. Our results are promising as control animals treated with PBS showed reduced 2D directional variance (enhanced alignment) of collagen fibers compared to animals treated with LXA_4_-ME, indicating that the collagen fibers were thicker and more parallel to each other, resembling scar tissue more than normal skin collagen fibers, confirming findings from other studies on scarring (42, 64).

The active resolution of inflammation promoted by SPMs impacts the same pro-inflammatory mediators that are the targets of pharmacologic inhibitors used for the management of several fibrotic diseases including corticosteroids (13, 65). The crucial difference is that a feed-forward, receptor-mediated response of active counter-regulation of pro-inflammatory signals (not inhibition) is coordinated temporally by SPMs and encompasses all relevant pathways, some of which may remain to be discovered. The mechanisms that underlie the positive therapeutic impact of LXA_4_-ME on scarring *via* modulating the density and alignment of collagen fibers merit further investigation. For instance, macrophages play a critical role in regulating wound healing (66). Decreasing the ratio of pro-inflammatory (M1-like) to pro-resolving (M2-like) macrophages promotes wound healing and scar resolution (67). Remarkably, *in vivo* administration of one SPM (RvD2) induced macrophages with unique pro-resolving properties stimulating inflammation resolution and muscle tissue regeneration (68) highlighting the potential of SPMs to favor wound healing by modifying the macrophage phenotype. In addition, T-cells derived from keloid scar tissue were abnormal compared with normal skin tissue (69). The positive therapeutic impact of LXA_4_-ME on scarring after cleft lip repair could be *via* modulating T-cell responses and decreasing their proinflammatory activities as it has been shown in other T-cell-mediated immune diseases (24). The mechanisms of SPMs on regulating the proliferation of fibroblasts, myofibrobasts and muscular cells to promote scarless wound healing are yet to be discovered.

## Study Limitations

While our data are robust and demonstrate consistent results on multiple different outcomes of interest (both subjective and objective), we must acknowledge study limitations. While the sample size used in our animal study was relatively small, the effect size of LXA_4_-ME was sufficient to provide power to detect a significant difference with 4 animals per group (32, 33).

## Conclusions

In summary, our data demonstrate that topical application of LXA_4_-ME reduces scar tissue formation, promotes tissue remodeling and improves wound healing after cleft lip repair surgery in rabbits. Our findings support the emerging therapeutic potential of LXA_4_-ME on scarring after cleft lip repair (**Figure 7**). Further translational research studies are needed to explore deeper the mechanisms of LXA_4_-ME improvement of healing outcomes after cleft lip repair surgeries.

## Data Availability Statement

The original contributions presented in the study are included in the article/**Supplementary Material**, further inquiries can be directed to the corresponding author.

## Ethics Statement

The animal study was reviewed and approved by Tufts University Institutional Animal Care and Use Committee (#B2017-58).

## Author Contributions

EP, contributed to the conception, design, animal experiments and interpretation of the data, drafted and critically revised the manuscript. CB and AS contributed to conception, design, part of the animal experiments, interpretation of the data, and critically revised the manuscript. CT and TVD contributed to conception, design, interpretation of the data, and critically revised the manuscript. LP, GH, YZ, and IG contributed to data analysis and their interpretation, and critically revised the manuscript. All authors gave final approval and agree to be accountable for all aspects of the work.

## Funding

Supported by USPHS grant K08DE027119 to EP, R01DE025020 to TVD for R01DE025020 and U01DE024503 to CT from the National Institute of Dental and Craniofacial Research (NIDCR) and National Institute of Health (NIH). Supported by NIH Research Infrastructure grant NIH S10 OD021624 to IG and by the National Center for Advancing Translational Sciences (NIH) UL1TR002544 to LP.

## Author Disclaimer

The content is solely the responsibility of the authors and does not necessarily represent the official views of the National Institutes of Health.

## Conflict of Interest

TVD is inventor on several granted and pending licensed and unlicensed patents awarded to the Forsyth Institute that are subject to consulting fees and royalty payments. TVD is a founder of Nocendra, Inc. and AIAH Inc.

The remaining authors declare that the research was conducted in the absence of any commercial or financial relationships that could be construed as a potential conflict of interest.

## Publisher’s Note

All claims expressed in this article are solely those of the authors and do not necessarily represent those of their affiliated organizations, or those of the publisher, the editors and the reviewers. Any product that may be evaluated in this article, or claim that may be made by its manufacturer, is not guaranteed or endorsed by the publisher.
