# Supplementary material for: Specialized Pro-Resolving Mediators Reduce Scarring After Cleft Lip Repair
Source: Front Immunol. 2022 Apr 27;13:871200. doi: 10.3389/fimmu.2022.871200 (PMC9094441; doi:10.3389/fimmu.2022.871200)
Supplement: Supplementary file 1 [file DataSheet_1.doc]

**APPENDIX**

**“Specialized Pro-resolving Mediators reduce**

**scarring after cleft lip repair.”**

Papathanasiou E ^1,2^, Scott AR ^3^, Trotman CA ^4^, Beale C ^5^, Price LL ^6,7^, Huggins G ^8^, Zhang Y ^9^, Georgakoudi I ^9^, Van Dyke TE ^2,10^.

**Supplemental Figure 1.** Modified Manchester Scar Scale

Supplemental Figure 2. Histologic Scar Assessment Scale


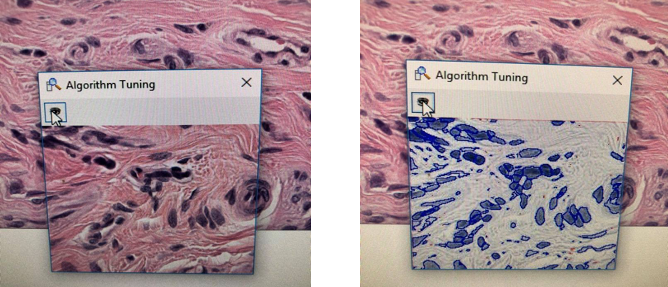


**Supplemental Figure 3.** Cell Nuclei Identification and Automatic Enumeration using the Nuclear image analysis algorithm (Nuclear V9) of Aperio Imagescope software. Using “algorithm tuning” in a mark-up window we first ensured that all nuclei are properly segmented and identified before enumerating them.


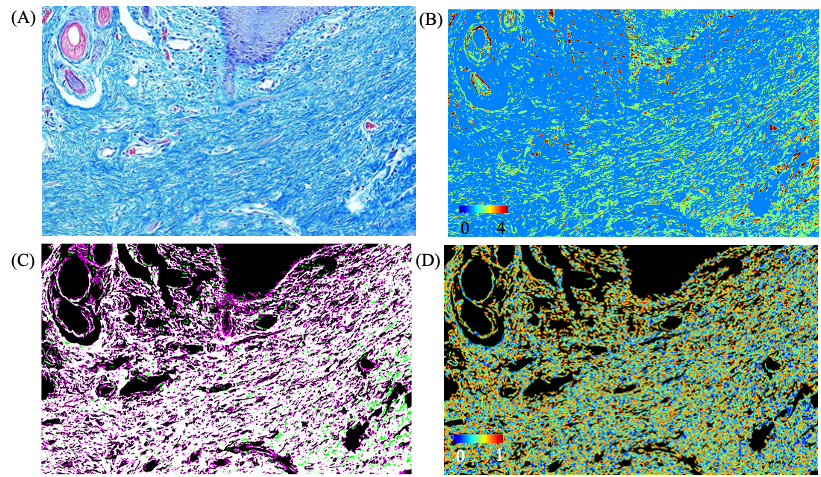


Supplemental Figure 4. RGB images from Masson’s Trichrome Stained tissue sections were used to assess collagen fiber density and orientation.

(A) An example of an RGB image from Masson’s Trichrome Stained tissue section. (B) In the B/G map of A, collagen has values that are smaller than 2.5 and those pixels are kept for the next thresholding step. (C) The overlay of the logical masks of the post-thresholded B/G map (green) and the post-thresholded R/B map (purple). Pixels with white hues (where the two masks overlap) are attributed to collagen and used for further analysis. (D) 2D directional variance map within a 3-pixel radius disk kernel filter.

Reliability of Scar Assessment Scales

It is important to determine reliability for our measurements for all scar assessment scales used in our preclinical study in an experimental model of cleft lip repair (Fig. 1) in order to optimize consistency and repeatability in our measurements and minimize random measurement errors. All scar assessment scales that were used in this preclinical experimental study showed good reliability and lack of evidence of systematic bias in grading scarring. The inter-rater reliability for two examiners (TVD, CAT) for Modified Manchester Scar was good with an ICC of 0.82 (95% CI=0.51 to 0.93,) (n=8, 1 image/rabbit), while the intra-rater reliability for our reference examiner (TVD) for Modified Manchester Scar was excellent with an ICC of 0.97 (95% CI=0.90 to 0.99) (n=8, 1 image/rabbit). Based on Bland-Altman plot and line of identity plot analyses (Supplemental Figures 5 and 6), there is lack of evidence of systematic bias in the reported Modified Manchester Scar scores.

The inter-rater reliability for two examiners (TVD, EP) for Histologic Scar assessment of 24 images (3 regions for 8 rabbits x 1 image/region) stained with H&E and Masson’s Trichrome was moderate with a range of weighted kappa statistics from 0.56-0.70 and 0.65-0.85, respectively. The intra-rater reliability for TVD for Histologic Scar assessment of 24 images (2 rounds of 3 regions for 8 rabbits x 1 image/region) stained with H&E and Masson’s Trichrome was good with a range of weighted kappa statistics from 0.67 to 1.00 and from 0.75 to 0.84, respectively.


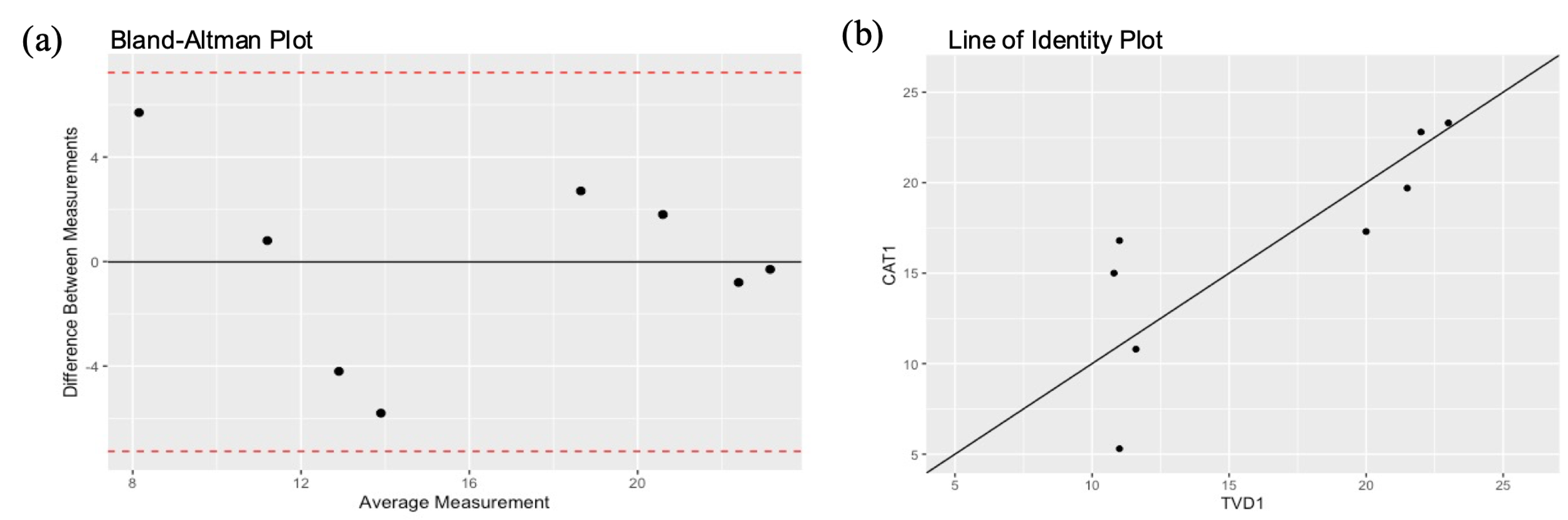


**Supplemental Figure 5.** Bland-Altman plot and Line of Identity plot for Modified Manchester Scar scores (inter-rater). (a) Bland-Altman plot with the differences in Modified Manchester Scar scores between two examiners plotted against their average measurements and representation of the 95% confidence limits of agreement (dotted lines) for two examiners (n=8). (b) Line of identity plot with Modified Manchester Scar scores from first round of grading from two examiners (n=8). (x=TVD1: Round 1 Dr. Van Dyke; y=CAT1: Round 1 Dr. Caroll Ann Trotman) (range of scale from 0-26).


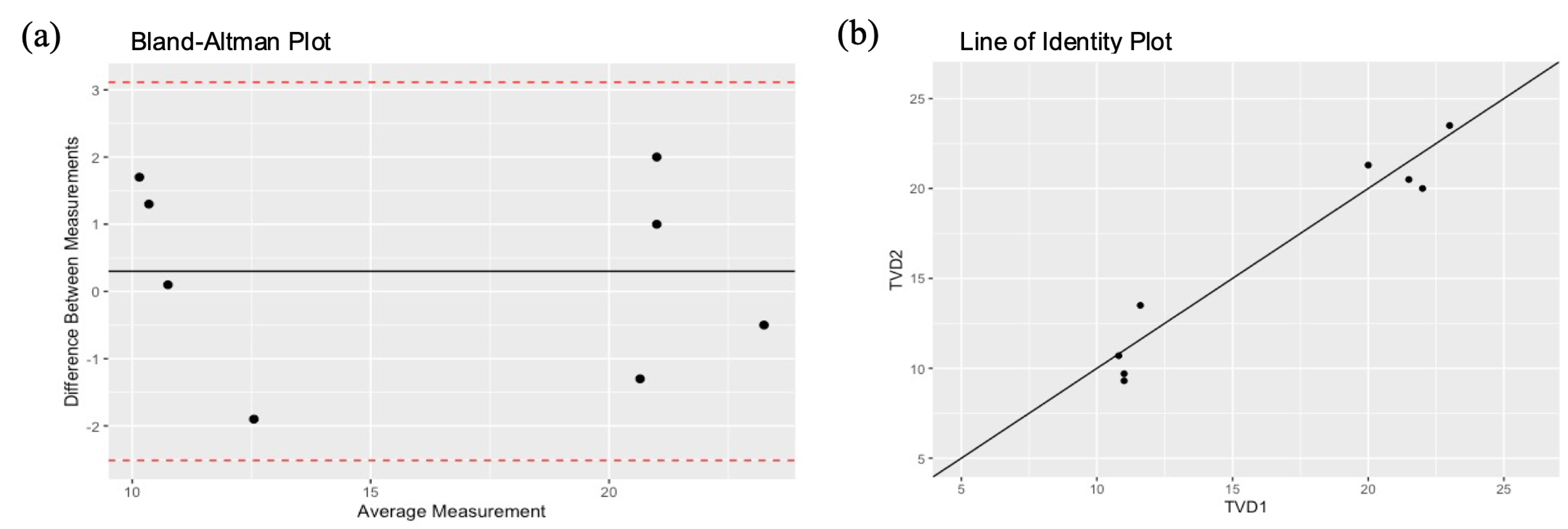


**Supplemental Figure 6.** Bland-Altman plot and Line of Identity plot for Modified Manchester Scar scores (intra-rater for TVD). (a) Bland-Altman plot with the differences in Modified Manchester Scar scores of between two rounds of grading from TVD plotted against their average measurements and representation of the 95% confidence limits of agreement (dotted lines) (n=8) (TVD=Dr. Thomas Van Dyke). (b) Line of identity plot with Modified Manchester Scar scores from two rounds of grading from TVD (n=8). (x=TVD1: Round 1 Dr. Van Dyke; y=TVD2: Round 2 Dr. Van Dyke) (range of scale from 0-26).
